# Supplementary figures and images for: Microencapsulation extends mycelial viability of Streptomyces lividans 66 and increases enzyme production
Source: BMC Biotechnol. 2018 Mar 12;18:13. doi: 10.1186/s12896-018-0425-2 (PMC5848461; doi:10.1186/s12896-018-0425-2)

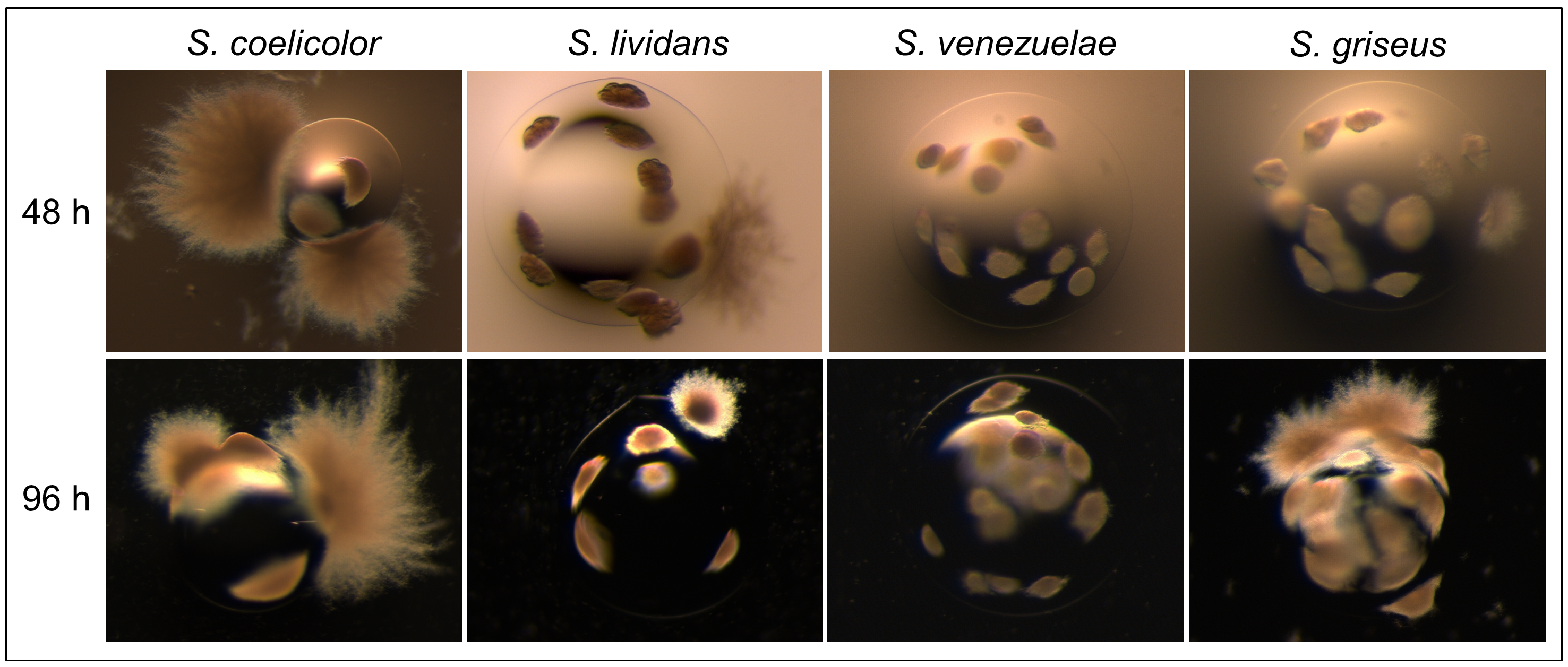

Supplement: Supplementary file 1 — Figure S1. Morphology of encapsulated streptomycetes in YEME medium. Microscopy images of microcapsules of Streptomyces coelicolor, Streptomyces lividans, Streptomyces venezuelae and Streptomyces griseus grown in YEME medium for 48 (top panel) and 96 h (lower panel). No scale bar is added since not all pictures are taken using the same magnification (mainly to allow the visualization of the protruding mycelium). As a reference, the microcapsules have an average size of 415 μm. (PNG 4150 kb) [file 12896_2018_425_MOESM1_ESM.png]

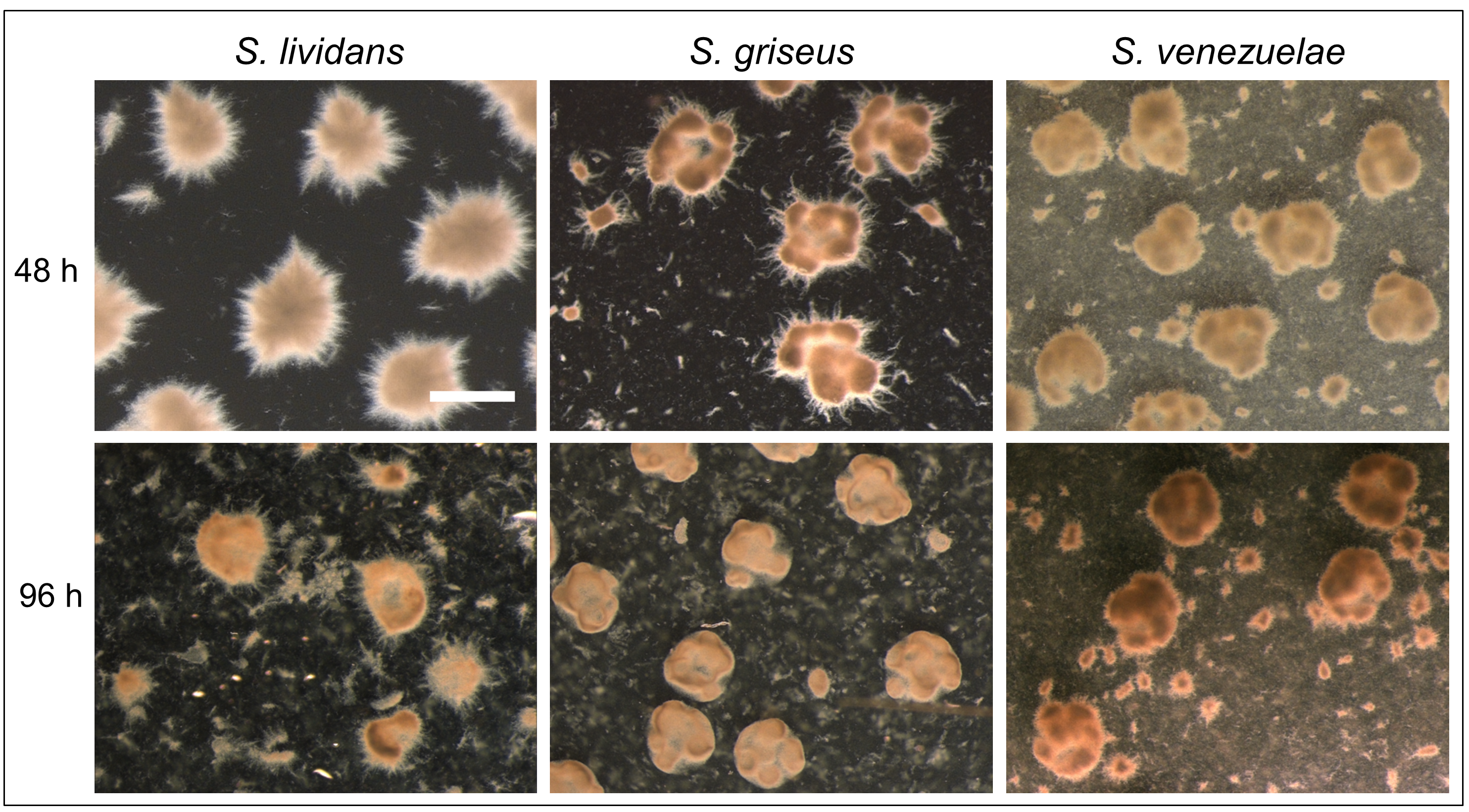

Supplement: Supplementary file 2 — Figure S2. Growth and detachment of mycelium from microcapsules containing different streptomycetes. Overview images of the mycelium of Streptomyces lividans, Streptomyces griseus and Streptomyces venezuelae grown in NMMPmod medium for 48 (top panel) and 96 h (lower panel). Note that detached mycelial fragments are evident in the culture broth of S. griseus and S. venezuelae at 48 h. After 96 h, detached mycelial fragments are also observed in S. lividans. The scale bar corresponds to 500 μm. (PNG 6146 kb) [file 12896_2018_425_MOESM2_ESM.png]

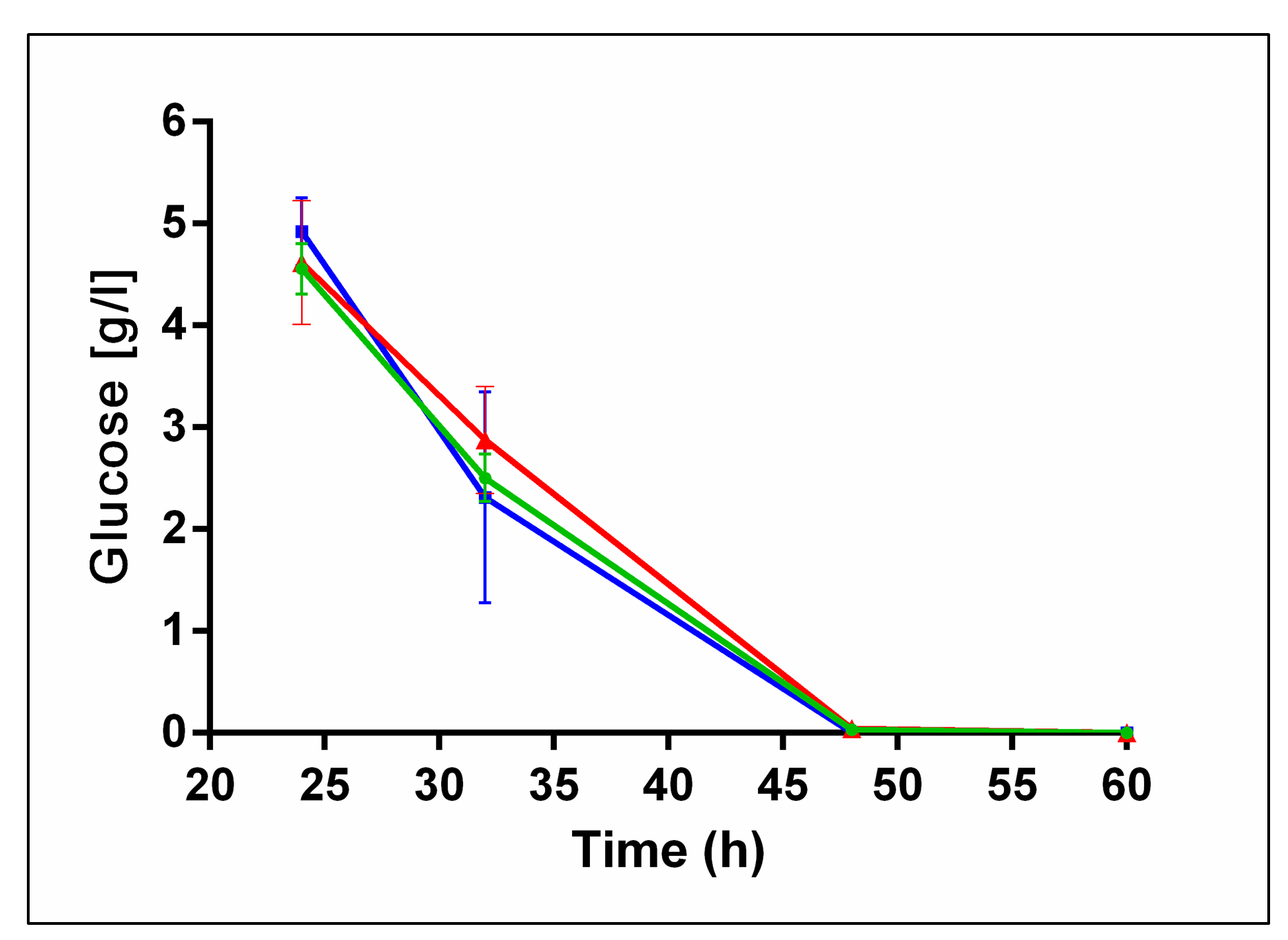

Supplement: Supplementary file 3 — Figure S3. Glucose consumption by encapsulated and non-encapsulated mycelium. The residual glucose concentrations (in g/L) in NMMPmod medium are shown when Streptomyces lividans pIJ703 is grown in micro-capsules (green), or non-encapsulated in the absence (red) and presence (blue) of a metal coil. (PNG 179 kb) [file 12896_2018_425_MOESM3_ESM.png]

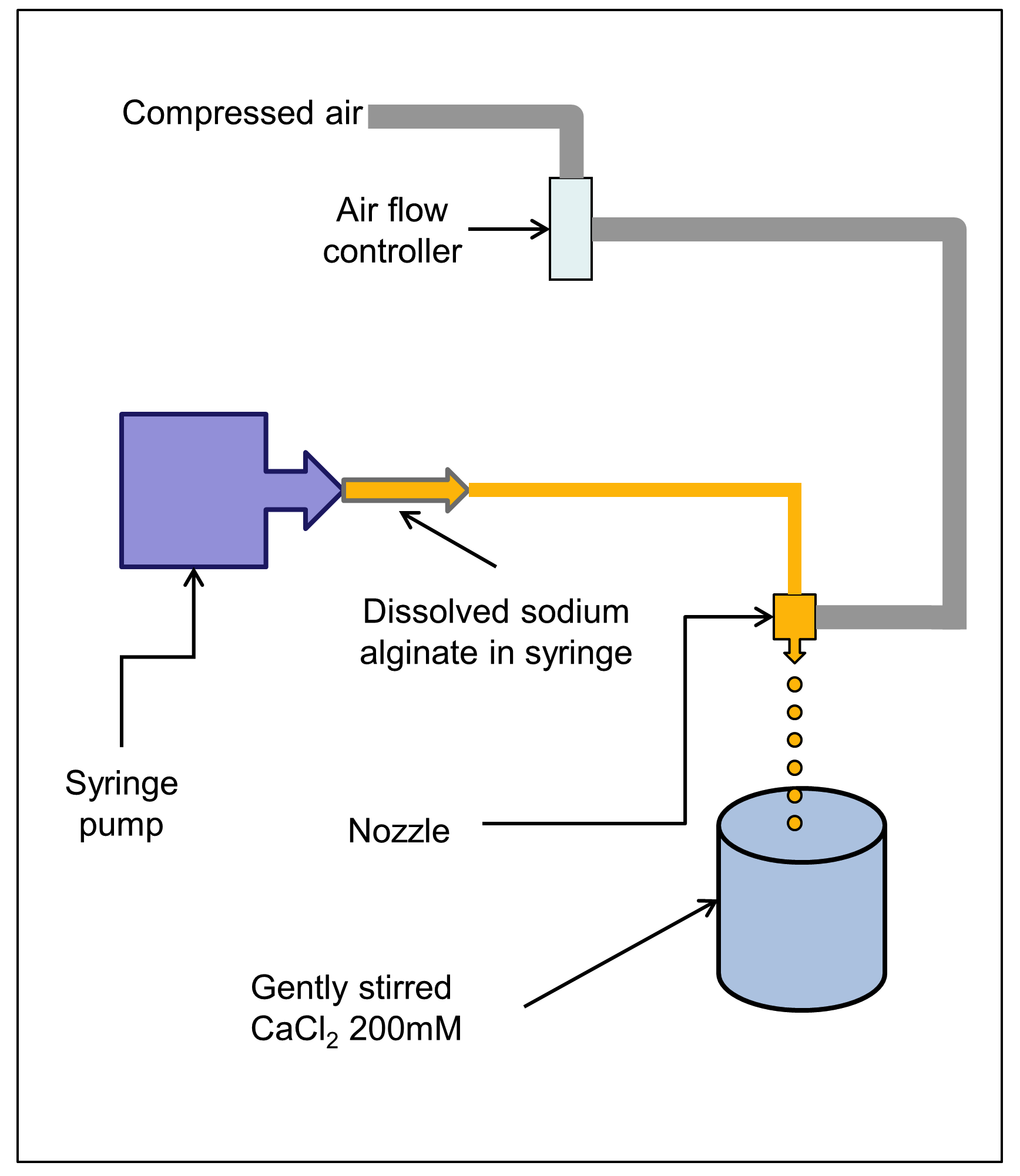

Supplement: Supplementary file 4 — Figure S4. Schematic representation of the microencapsulation apparatus. A constant flow of dissolved alginate is maintained with a syringe pump set at 30 ml h− 1, while the air flow is regulated with an air flow controller set at 3 L h− 1. The sodium alginate droplets that detach form the nozzle fall in a gently-stirred solution of 200 mM CaCl2, leading to formation of the microcapsules via ion exchange. The nozzle functions according to the coaxial gas-flow extrusion principle [33]. (PNG 155 kb) [file 12896_2018_425_MOESM4_ESM.png]
